# Spectrum of Disorders Associated with Elevated Serum IgG4 Levels Encountered in Clinical Practice

**DOI:** 10.1155/2012/232960

**Published:** 2012-05-27

**Authors:** Jay H. Ryu, Ryohei Horie, Hiroshi Sekiguchi, Tobias Peikert, Eunhee S. Yi

**Affiliations:** ^1^Division of Pulmonary and Critical Care Medicine, Mayo Clinic, Gonda 18 South, 200 First St. SW, Rochester, MN 55905, USA; ^2^Division of Anatomic Pathology, Mayo Clinic, Rochester, MN 55905, USA

## Abstract

IgG4-related disease (IgG4-RD) is a recently described systemic fibroinflammatory disease associated with elevated circulating levels of IgG4 and manifests a wide spectrum of clinical presentations. Although serum IgG4 level has been described to be the most sensitive and specific laboratory test for the diagnosis of IgG4-RD, it is recognized that an elevated serum IgG4 level can be encountered in other diseases. In this study, we sought to identify the frequency of IgG4-RD and other disease associations in patients with elevated serum IgG4 levels seen in clinical practice. Among 3,300 patients who underwent IgG subclass testing over a 2-year period from January 2009 to December 2010, 158 (4.8%) had an elevated serum IgG4 level (>140 mg/dL). IgG4 subclass testing was performed for evaluation of suspected IgG4-RD or immunodeficiency. Twenty-nine patients (18.4%) had definite or possible IgG4-RD. Among those patients without IgG4-RD, a broad spectrum of biliary tract, pancreatic, liver, and lung diseases, as well as systemic vasculitis, was diagnosed. We conclude that patients with elevated serum IgG4 levels encountered in clinical practice manifest a wide array of disorders, and only a small minority of them has IgG4-RD.

## 1. Introduction

IgG4-related disease (IgG4-RD) is a recently described systemic fibroinflammatory disease associated with elevated circulating levels of IgG4 [1–4]. The pathologic lesion of IgG4-RD is characterized by lymphoplasmacytic inflammation with increased numbers of IgG4-positive plasma cells, fibrosis, and phlebitis. Although initial descriptions of this disorder focused on its pancreatic presentation (autoimmune pancreatitis), it has become apparent that IgG4-RD is a systemic disease associated with a wide spectrum of clinical manifestations involving virtually any organ in the body.

As initially observed in patients with autoimmune pancreatitis, the majority of patients with IgG4-RD have an elevated serum IgG4 level. Although serum IgG4 level has been described to be the most sensitive and specific laboratory test for the diagnosis of IgG4-RD, it is recognized that an elevated serum IgG4 level can be encountered in other diseases such as pancreatic cancer [5], atopic diseases [6], and infections [7, 8]. Furthermore, serum IgG4 level is elevated in up to 5% of the normal population [9, 10]. In this study, we sought to identify the spectrum of diseases associated with elevated serum IgG4 levels in patients encountered in clinical practice and the frequency of IgG4-RD in this population.

## 2. Methods

Using a computer-assisted search, we identified all patients who had serum IgG subclass levels determined on one or more occasions at Mayo Rochester, MN, USA, during the 2-year period from January 1, 2009 to December 31, 2010 and selected those with an elevated serum IgG4 concentration (>140 mg/dL) for analysis [10]. The concentrations of IgG subclass proteins in serum were measured in the Mayo Clinic Clinical Laboratory by automated nephelometry in which the concentrations of each protein were determined from standard curves [11]. Human IgG4 latex reagent (Binding Site Group Ltd, Birmingham, UK) was used in quantifying the serum IgG4 concentration. Medical records of those patients with elevated serum IgG4 levels were reviewed to extract data regarding age, sex, clinical presentation, serum IgG4 level, indication for serum IgG subclass determination, imaging and biopsy results, and diagnoses. The main diagnosis that resulted from evaluation of the presenting clinical issue at the time of the serum IgG subclass testing was identified.

For this study, the diagnostic criteria similar to those proposed by Umehara and colleagues were employed [12]. The “definite” diagnosis of IgG4-RD required the following criteria in addition to the known serum IgG4 elevation: (1) clinical and/or radiologic evidence of lesions consistent with IgG4-RD in one or more organs as previously described in the literature and (2) IgG4 staining showing greater than 10 IgG4^+^ cells/high-power field and IgG4^+^/IgG^+^ ratio greater than 40% in the presence of lymphoplasmacytic infiltration and fibrosis. Patients who fulfill the organ-specific criteria for IgG4-related autoimmune pancreatitis, IgG4-related Mikulicz's disease, and IgG4-related kidney disease were also designated as having “definite” IgG4-RD [12]. Those patients clinically diagnosed based on clinical and imaging features along with an elevated serum IgG4 level but not fulfilling histopathologic criteria outlined above or in the absence of tissue biopsy and exhibiting improvement with corticosteroid therapy were designated as “possible” IgG4-RD in the absence of any other more likely diagnoses. None of our patients met the criteria for “probable” IgG4-RD outlined by Umehara and colleagues [12]. Non-IgG4-RD diagnoses were determined based on the results of the diagnostic evaluation, the clinicians' diagnostic impression, and the subsequent clinical course. Approval was obtained from the Mayo Foundation Institutional Review Board prior to beginning the study.

### 2.1. Statistical Analysis

Data are presented as mean ± SD and percentages for categorical variables unless stated otherwise. Demographic data were compared using the Fisher's exact test. Means of continuous variables were compared between groups with a two-sample *t-*test. Serum IgG4 levels between groups were compared using the Wilcoxon rank-sum test. In all cases *P*-values <0.05 were considered statistically significant.

## 3. Results

We identified 3,300 consecutive patients who had their serum IgG subclass testing performed on one or more occasions during the 2-year interval from January 1, 2009 to December 31, 2010; 158 patients (4.8%) had at least one high serum IgG4 level (>140 mg/dL). The demographic features and serum IgG4 level of these 158 patients are outlined in Table 1. Indications for IgG subclass testing were evaluation for possible IgG4-RD in 104 patients (65.8%) and to assess for immunodeficiency (e.g., patients with recurrent or chronic infections) in 54 patients (34.2%).

**Figure 1:**
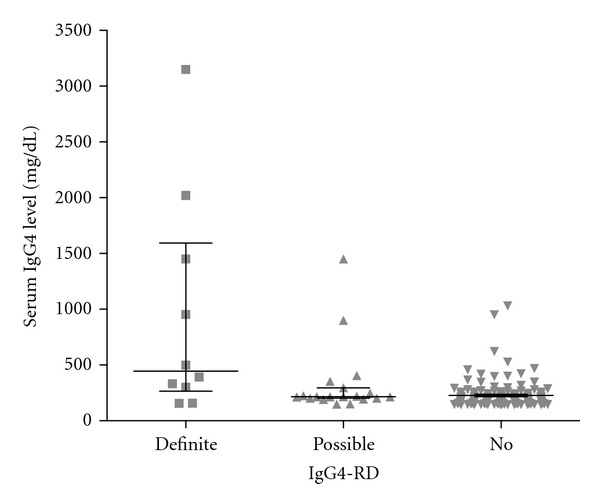
Serum IgG4 levels for definite IgG4-RD, possible IgG4-RD, and non-IgG4-RD subgroups. Median and interquartile range (25% to 75%) are depicted.

**Figure 2:**
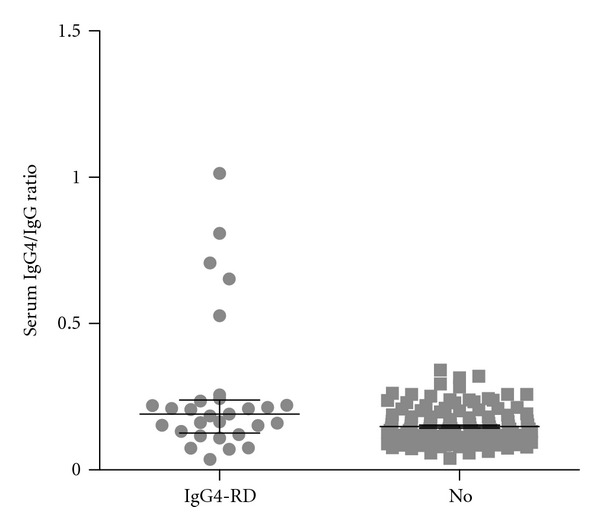
Serum IgG4/IgG ratios for IgG4-RD (definite and possible IgG4-RD) and non-IgG4-RD subgroups. Median and interquartile range (25% to 75%) are depicted.

Twenty-nine patients (18.4%) met the criteria for definite or possible IgG4-RD (Table 2). The mean age of those with IgG4-RD was older compared to those without IgG4-RD (58.3 ± 16.9 versus 49.9 ± 20.8, *P* < 0.05) but the gender distribution was not different (*P* = 0.29). The serum IgG4 level was significantly higher in those with IgG4-RD compared to those with non-IgG4-RD diagnoses (*P* < 0.001) (Table 1). Furthermore, mean serum IgG4 level was higher for those with definite IgG4-RD (940 ± 990) compared to possible IgG4-RD (329 ± 318) which, in turn, was higher compared to non-IgG4-RD subgroup (226 ± 127) (*P* < 0.05) (Figure 1). The mean serum IgG4/IgG ratio was significantly higher in patients with IgG4-RD (definite and possible) compared to non-IgG4-RD patients (0.263 ± 0.239 versus 0.148 ± 0.061, *P* < 0.01), but there was substantial overlap in individual values between the two groups as shown in Figure 2. Among 29 patients with IgG4-RD, 10 patients met the criteria for definite IgG4-RD which included supportive histopathologic findings on tissue biopsy. Of 19 patients with possible IgG4-RD, 12 had undergone biopsy procedures but the biopsy findings did not meet the criteria for definite or probable IgG4-RD diagnosis.

In the remaining 129 patients who did not have IgG4-RD, the most common diagnoses were primary sclerosing cholangitis, bronchiectasis, non-IgG4-related pancreatitis, vasculitis, chronic rhinosinusitis, and pancreatic or bile duct cancer. No specific diagnosis was achieved in 29 patients; most common presenting complaints in these patients included abdominal pain, fevers, and lymphadenopathy.

**Figure 3:**
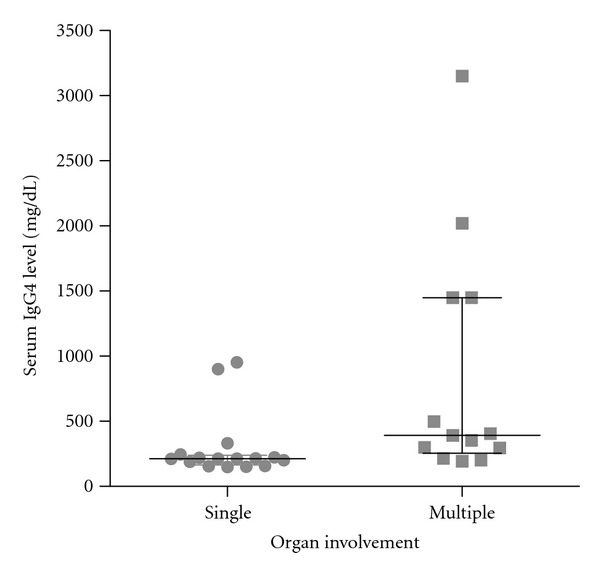
Serum IgG4 levels for patients with single-organ and multiple-(two or more) organ involvement. Median and interquartile range (25% to 75%) are depicted.

Pattern of organ involvement for these patients with IgG4-RD is outlined in Table 3. Pancreas, bile ducts, and orbital structures were most commonly involved. However, these patients exhibited involvement in a number of other organs including the salivary glands, retroperitoneal region, lymph nodes, kidney, lung, pleura, sinuses, gastrointestinal tract, and testis. Patients with two or more organ involvements (*n* = 13) had a higher mean serum IgG4 level (840 ± 914) compared to those with single organ involvement (296 ± 250) (*P* < 0.01) (Figure 3).

All except one patient (surgical resection of a biliary lesion) with IgG4-RD (definite or possible) were treated with prednisone and improved. Twelve of 28 patients initially treated with prednisone experienced subsequent relapses requiring reinstitution of prednisone alone or in combination with another immunosuppressive agent (including azathioprine, methotrexate, and rituximab). Median duration of followup was 23 months (range, 1 to 126 months).

## 4. Discussion

In this study we found a broad spectrum of diagnoses to be associated with elevated serum IgG4 levels encountered in the clinical practice setting; less than one-fifth of these patients manifested evidence for IgG4-RD. IgG4 subclass testing was ordered by clinicians for evaluation of possible IgG4-RD or immunodeficiency, and thus it is not surprising that various types of non-IgG4-related pancreatic and biliary tract disorders were included along with chronic infections, for example, bronchiectasis and sinusitis.

At the present time, there is no published international consensus on the diagnostic criteria for IgG4-RD. Most authors agree that definitive diagnosis of IgG4-RD requires histologic confirmation that includes the presence of characteristic histopathologic features (lymphoplasmacytic infiltration, fibrosis, and obliterative phlebitis or arteritis) along with immunostaining that demonstrates increased numbers of IgG4^+^ cells. Various authors have used different cutoffs for IgG4 staining criteria. For the purposes of this study, we used the diagnostic criteria recently published by Umehara and colleagues [12] for “definite,” “probable,” and “possible” IgG4-RD cases. In clinical practice, there are patients in whom the diagnosis of IgG4-RD is likely and are empirically treated without biopsy confirmation. This may occur when patients decline invasive procedures or the initial biopsy specimen is nondiagnostic and additional biopsies are not pursued for based on patient preference, perceived risks, or lack of any other likely diagnosis.

All patients included in this analysis exhibited elevated serum IgG4 levels and the degree of this elevation was not used in determining the presence or absence of IgG4-RD. Our patients with IgG4-RD exhibited a significantly higher serum IgG4 levels compared to those without IgG4-RD although there was an overlap in their serum IgG4 values. Some authors have suggested that a serum IgG4 concentration that is more than twice the upper limit of normal (>280 mg/dL) is highly specific for IgG-RD [13]. In this study 13 of 29 patients (45%) with IgG4-RD and 18 of 129 patient (14%) without IgG4-RD had a serum IgG4 concentration that was >280 mg/dL.

The sensitivity of elevated serum IgG4 levels in the diagnosis of IgG4-RD has been reported to be in the range of 67% to 95% and specificity to be 90% to 97% [1, 2, 10, 13–17]. Serum IgG4 elevation is present in 5% of the normal population [9, 10] and has been observed in patients with other disorders. For example, serum IgG4 levels have been reported to be elevated in 7% to 10% of patients with pancreatic cancer [5, 13]. Similarly, serum IgG4 elevation has been seen in 5% to 9% of patients with other forms of pancreatitis and benign pancreatic tumors [13]. Similar spectrum of pancreatic diseases was also seen in our study cohort.

Nonpancreatic disorders have also been associated with elevated serum IgG4 levels. These include skin diseases including atopic dermatitis [6] and pemphigus vulgaris [18, 19], as well as parasitic diseases [7, 8]. Our study cohort included one patient with atopic dermatitis, but none had pemphigus or parasitic diseases.

One-fifth of our cohort had various respiratory diseases. Van Nieuwkoop and colleagues [20] had previously described an association between a polyclonal increase in serum IgG4 subclass with acquired respiratory diseases. Bronchiectasis and chronic rhinosinusitis were most respiratory disorders in our study cohort likely reflecting the fact that suspected immunodeficiency was one of the main indications for IgG subclass testing in this population.

Primary sclerosing cholangitis (PSC) was the single most common diagnosis in the non-IgG4-RD group. Nine of 17 patients with PSC had underlying inflammatory bowel disease. Elevated serum IgG4 levels have been described in 9% to 12% of patients with PSC [21, 22]. Since differentiation of autoimmune pancreatitis from pancreatic cancer and biliary tract disease is a common clinical indication for IgG subclass testing, it seems reasonable to expect that patients with various types of biliary tract diseases including PSC and cholangiocarcinoma will be encountered in those patients with elevated serum IgG4 levels.

Vascular involvement has been seen in IgG4-RD mainly in the form of aortitis, periaortitis, and inflammatory abdominal aortic aneurysm [2, 23, 24]. Recently, elevated serum IgG4 levels have been reported in patients with Churg-Strauss syndrome [25, 26] and hypocomplementemic urticarial vasculitis [27]. Additionally, IgG4 antiproteinase 3 autoantibodies have been demonstrated to stimulate neutrophils to undergo a proinflammatory response suggesting potential relevance in the pathogenesis of granulomatosis with polyangiitis (Wegener's) [28]. In this regard, it is interesting to note that nine patients in our study cohort had systemic vasculitis including granulomatosis with polyangiitis and the Churg-Strauss syndrome. Prevalence of high serum IgG4 level in patients with ANCA-associated vasculitis and the relevance of IgG4 in the pathogenesis of these disorders need to be explored further.

Although IgG4-related hepatopathy and hepatic inflammatory pseudotumors have been described [29–31], none of our patients with liver disease and elevated serum IgG4 levels fulfilled the criteria for IgG4-RD. They had other identifiable causes including hepatitis C, drugs, sarcoidosis, and primary biliary cirrhosis.

As previously noted, serum IgG4 level is elevated in 5% of the normal population [9, 10]. Similarly, 4.8% of 3,300 consecutive patients undergoing serum IgG subclass testing at our institution over a 2-year period exhibited an elevated serum IgG4 level. It seems reasonable to assume that elevated serum IgG4 level will be found coincidentally in a small portion of various disease populations that are subjected to IgG subclass testing, for example, patients with bronchiectasis. None of our 54 patients with high serum IgG4 level who had undergone IgG subclass testing for the indication of suspected immunodeficiency had evidence of IgG4-RD. It appears unlikely that there is a causal relationship between the elevated serum IgG4 level and many of the diseases listed in Table 2. It remains to be determined whether elevated serum IgG4 level is a relevant finding in other disorders other than those recognized currently as IgG4-RD.

There are several limitations to this study. This study was a retrospective survey with analysis limited to the clinical data available in medical records and imaging studies. The diagnostic evaluation for these patients was performed by various clinicians at our institution according to their own clinical judgment and patient context. It is possible that some cases of IgG4-RD may have been missed particularly in those patients without a specific diagnosis. In addition, the extent of organ involvement may have been underestimated in patients with IgG4-RD due to lack of relevant imaging studies or biopsy specimens in the absence of standard methodical evaluation.

We conclude that only a minority of patients with elevated serum IgG4 levels encountered in clinical practice have IgG4-RD. Furthermore, elevated serum IgG4 levels can be seen in patients with many different diseases, most of which likely represent coincidental occurrence. Our findings reinforce the principle that an elevated serum IgG4 level in isolation is of limited diagnostic utility.

## Figures and Tables

**Table 1:** Demographics and serum IgG4 levels (*n* = 158).

Characteristic	All patients	IgG4-RD (*n* = 29)	No IgG4-RD (*n* = 129)
Age, yr	51.4 ± 20.3	58.3 ± 16.9	49.9 ± 20.8
Male sex, no. (%)	98 (62)	21 (72)	77 (60)
Serum IgG4 level, mg/dL, median (range)	202 (141–3150)	223 (149–3150)	187 (141–1030)

Data are presented as mean ± SD, unless otherwise indicated.

IgG4-RD: IgG4-related disease.

IgG4-RD includes definite and possible cases.

**Table 2:** Spectrum of diagnoses associated with high serum IgG4 Levels (*n* = 158).

Diagnosis	No. of patients (%)
IgG4-related disease	29 (18.4)
Definite	10
Probable	0
Possible	19
Respiratory diseases	32 (20.3)
Bronchiectasis	11
Chronic rhinosinusitis	7
Asthma	4
Idiopathic pulmonary fibrosis	3
Sarcoidosis	2
Other respiratory diseases*	5
Biliary tract diseases	26 (16.5)
Primary sclerosing cholangitis	17
Cholangiocarcinoma	6
Biliary stricture or stone	3
Pancreatic diseases	19 (12.0)
Pancreatitis, not IgG4 related	10
Pancreatic cancer	6
Other pancreatic diseases^†^	3
Cirrhosis and other liver diseases	9 (5.7)
Vasculitis	9 (5.7)
Granulomatosis with polyangiitis (Wegener's)	5
The Churg-Strauss syndrome	3
Polyarteritis nodosa	1
Atopic dermatitis	1 (0.6)
Miscellaneous diseases^‡^	4 (2.5)
No specific diagnosis	29 (18.4)

*Other respiratory diseases (5 patients) included chronic pleuritis, emphysema, fibrosing mediastinitis, hypersensitivity pneumonitis, and recurrent pneumonias, respectively.

^†^Other pancreatic diseases (3 patients) included intraductal papillary mucinous neoplasm, pancreatic cyst, and pancreatic insufficiency, respectively.

^‡^Miscellaneous diseases (4 patients) included lactose intolerance, neurofibromatosis, polymyositis, and psoriasis, respectively.

**Table 3:** Spectrum of organ involvement in patients with IgG4-RD (*n* = 29).

Organ	Definite cases, *n* (*n* = 10)	Possible cases, *n* (*n* = 19)	All cases, *n* (%)
Pancreas	6	12	18 (62)
Bile ducts	2	9	11 (38)
Orbit	2	2	4 (14)
Retroperitoneum	1	2	3 (10)
Salivary glands	2	1	3 (10)
Lymph nodes	1	1	2 (7)
Lung	1	1	2 (7)
Gastrointestinal	1	1	2 (7)
Kidney	1	0	1 (3)
Pleura	1	0	1 (3)
Sinus	1	0	1 (3)
Mesentery	0	1	1 (3)
Testis	1	0	1 (3)
